# Association of FOXO3 Expression with Tumor Pathogenesis, Prognosis and Clinicopathological Features in Hepatocellular Carcinoma: A Systematic Review with Meta-Analysis

**DOI:** 10.3390/cancers13215349

**Published:** 2021-10-26

**Authors:** Flavia Fondevila, Paula Fernández-Palanca, Carolina Méndez-Blanco, Tania Payo-Serafín, Elisa Lozano, Jose J. G. Marin, Javier González-Gallego, José L. Mauriz

**Affiliations:** 1 Institute of Biomedicine (IBIOMED), University of León, Campus de Vegazana s/n, 24071 León, Spain; ffonp@unileon.es (F.F.); pferp@unileon.es (P.F.-P.); cmenb@unileon.es (C.M.-B.); tpayos00@estudiantes.unileon.es (T.P.-S.); jgonga@unileon.es (J.G.-G.); 2 Centro de Investigación Biomédica en Red de Enfermedades Hepáticas y Digestivas (CIBERehd), Instituto de Salud Carlos III, Av. de Monforte de Lemos 5, 28029 Madrid, Spain; elisa_biologia@usal.es (E.L.); jjgmarin@usal.es (J.J.G.M.); 3 Experimental Hepatology and Drug Targeting (HEVEPHARM), Salamanca Biomedical Research Institute (IBSAL), University of Salamanca, 37007 Salamanca, Spain

**Keywords:** clinicopathological features, diagnosis, forkhead box O3, hepatocellular carcinoma, prognosis

## Abstract

**Simple Summary:**

Liver cancer, mainly represented by hepatocellular carcinoma (HCC), constitutes the current third leading cause of tumor-associated death worldwide. Therefore, finding new molecules that improve early HCC diagnosis, prognosis and patient outcomes is crucial. Forkhead box O3 (FOXO3), a central factor expressed by hepatocytes, has been related to cancer progression. This novel systematic review, with meta-analysis, aimed to unravel the diagnostic and prognostic value of FOXO3 expression in HCC. We systematically searched Cochrane, Embase, PubMed, Scopus and Web of Science for articles evaluating FOXO3 levels in HCC samples and its association with HCC development, survival or clinicopathological features. After study selection, overall effect and heterogeneity assessment, and subgroup and publication bias analysis were carried out. Based on five studies involving 1059 cases, we found that high FOXO3 expression correlates with tumor development, poor survival and invasion in HCC. Thus, FOXO3 emerges as a novel diagnostic and prognostic biomarker for HCC monitoring.

**Abstract:**

Forkhead box O3 (FOXO3), an essential transcription factor related to liver disease, has been linked to cancer progression. The most frequent primary liver tumor, hepatocellular carcinoma (HCC), has an elevated mortality rate and patient outcomes remain very poor. Here, we examined the diagnostic, prognostic and clinicopathological significance of FOXO3 expression in HCC. We systematically searched Cochrane, Embase, PubMed, Scopus and Web of Science. Articles analyzing FOXO3 levels in HCC patient samples and its relationship with tumor development, survival or clinicopathological factors were selected. Hazard ratios, odds ratios and 95% confidence intervals were extracted, estimated by Parmar method or calculated and pooled across studies. Heterogeneity was evaluated by chi-square-based Q and I^2^ tests, while publication bias by funnel plots and Egger’s test. Subgroup analysis was performed when heterogeneity was evident. The study protocol was registered in PROSPERO (CRD42021237321), and data were meta-analyzed employing STATA 16. Five studies involving 1059 HCC cases were finally included in this meta-analysis, finding that high FOXO3 levels significantly correlate with HCC development and shorter overall survival. Moreover, subgroup analysis revealed a significant association between positive FOXO3 expression and the risk of invasion. Thus, FOXO3 could function as a novel biomarker with diagnostic and prognostic value in HCC.

## 1. Introduction

Liver cancer constitutes a major health problem worldwide, ranking as the sixth most frequently diagnosed cancer and the third leading cause of cancer-associated death [1]. Among primary liver tumors, approximately 85% of cases are hepatocellular carcinoma (HCC) [1,2], an aggressive malignancy with high incidence and mortality rates [3,4,5]. Since the majority of HCC cases are detected at advanced stages [3,4], only a slight percentage of patients are eligible for curative therapeutical approaches and, unfortunately, post-operative relapse is frequent [2,3]. Despite the latest advances in diagnostic and therapeutic strategies, the prognosis of HCC remains very poor, with an overall 5-year survival rate lower than 18% [6]. Thus, there is an imperative need to identify novel biomarkers able to optimize early diagnosis, guide treatment application and improve patients’ survival.

The forkhead box (FOX) family is organized into 19 different sub-families of transcription factors that share a highly conserved DNA-binding domain named forkhead box or winged-helix domain. In mammals, the O subgroup (FOXO) is composed of FOXO1, FOXO3, FOXO4 and FOXO6 [7,8,9]. Specifically, FOXO3 has shown to play physiological and pathological roles by controlling the transcription of target genes involved in oxidative defense, metabolic state [9,10], proliferation, cell cycle, autophagy [10] and apoptosis [10,11]. However, there are opposing reports about the role of FOXO3 expression in cancer, finding that FOXO3 upregulation can act either as a tumor-suppressive or an oncogenic mechanism, depending on the tumor type or specific circumstances [7,10].

Although FOXO3 is ubiquitously expressed [8,10], hepatocytes usually exhibit high levels [7], and this transcription factor seems to be implicated in the pathogenesis of liver disease [12]. Regarding liver cancer, several studies sustain that abnormal FOXO3 overexpression could represent an unfavorable hallmark in HCC, being linked to multidrug resistance [13,14], more aggressive phenotypes and a worse long-term prognosis [12,15,16,17]. Conversely, other investigations defend the correlation of low levels of FOXO3 with the development of chemoresistance [18] and poorer HCC patient outcomes [19,20]. Therefore, existing HCC articles dealing with the association of FOXO3 expression with survival parameters and clinicopathological characteristics show controversial results, being necessary to clarify the role played by FOXO3 in HCC.

Here, we conducted the first systematic review with meta-analysis aimed to explore the relationship of FOXO3 expression with HCC pathogenesis, clinicopathological features and patient outcomes. Thus, we sought to contribute to a deeper understanding of HCC and provide a novel molecule with diagnostic and prognostic value, enabling better diagnosis and patient surveillance. Although further investigations are required, our systematic review with meta-analysis demonstrated the promising value of FOXO3 as a novel biomarker for HCC, revealing that high FOXO3 expression is significantly correlated with tumor development, shorter survival rate and the risk of invasion.

## 2. Materials and Methods

### 2.1. Study Objectives

In the current investigation, we aimed at analyzing the diagnostic and prognostic value of FOXO3 expression in patients with HCC, focusing on the association of FOXO3 expression with tumor development and survival parameters, as well as with several tumor and patient characteristics.

This meta-analysis was performed following the Preferred Reporting Items for Systematic Reviews and Meta-Analyses (PRISMA) guidelines (Tables S1 and S2) [21]. Moreover, the study protocol was previously registered in the International Prospective Register of Systematic Reviews (PROSPERO), being assigned the CRD42021237321 registration code.

### 2.2. Literature Search Strategy

An exhaustive literature search was accomplished in Cochrane, Embase, PubMed, Scopus and Web of Science (WOS) databases, establishing 30th April 2021 as the inclusion deadline date. The eligible studies were identified by employing the search strategies indicated in Table 1.

### 2.3. Inclusion and Exclusion Criteria

We selected the articles satisfying the following criteria: (1) studies involving patients diagnosed with HCC; (2) determination of FOXO3 expression at translational or transcriptional level in tumor tissue; (3) relationship of FOXO3 levels with tumor presence/absence, survival data or clinicopathological features reported.

Studies complying with the following criteria were excluded: (1) studies exclusively accomplished with pre-clinical models; (2) reviews, book chapters, meeting communications and similar articles; (3) studies without mandatory data directly provided or in which it cannot be estimated; (4) articles with no available full-text in English.

### 2.4. Data Extraction and Quality Assessment

Articles screening, as well as data extraction and quality assessment from each included study, was independently performed by four researchers. Discrepancies were solved by discussion and final consensus.

Determination of the quality of selected articles was carried out using the Newcastle–Ottawa scale (NOS), which scores studies from 0 to 9 [22]. Only high-quality studies (NOS score ≥ 5) were included in the quantitative synthesis, whereas studies that scored < 5 were considered as low-quality and were omitted.

Baseline characteristics from each selected article are recorded in Table 2, which recapitulates the following information: study, year of publication, tumor sample size, gender, patients’ origin, intervention, pre- or post-surgery treatment, age range, mean/median age, study quality, method by which FOXO3 expression was measured, survival analysis performed, source of hazard ratio (HR), healthy liver sample size, definition of high FOXO3 expression and number of tumor or healthy liver samples with such high FOXO3 expression. Furthermore, the antibodies and the staining procedure used by the included articles evaluating FOXO3 levels through immunohistochemistry (IHC) are collected in Table S3.

### 2.5. Statistical Analysis

Meta-analysis synthesis was conducted using the STATA software version 16 (Stata Corporation, College Station, TX, USA).

We examined the role of FOXO3 expression on HCC patients in two steps. In the first step, we pooled the overall survival (OS) by HR and 95% confidence interval (CI), to calculate the effective value and unravel the correlation between FOXO3 and HCC patients’ life expectancy. OS was established as the time from the intervention date until the day of decease or the last follow-up visit. Parmar method [23] was employed to estimate these data when no explicit information was reported in the primary study. HRs and respective 95% CIs were combined throughout the studies. In the second step, the power of the association of FOXO3 overexpression with tumor existence or clinicopathological features was estimated by odds ratio (OR) with 95% CI. Besides, specific thresholds or cut-off values were established to calculate the possible correlation between FOXO3 high levels and certain clinicopathological factors: alpha-fetoprotein (AFP), 50 ng/mL; tumor-node-metastasis (TNM) staging, I-II/III-IV; tumor size, 5 cm. Combined HR > 1 and OR > 1 denoted an upper risk of poor prognosis and a higher incidence of the tested feature when high expression of FOXO3, respectively, considering significant when *p* < 0.05.

Heterogeneity was assessed by chi-square-based Q-test and I^2^ statistic, an indicator of inconsistency across studies that ranges from 0% (no observed heterogeneity) to 100% (maximal heterogeneity). The Restricted Maximum Likelihood (REML) method was used as the random-effect model in cases where heterogeneity was detected (Q-test *p*-value < 0.10 and/or I^2^ ≥ 50%). Otherwise, the fixed-effects model with Inverse Variance (IV) method was employed [24]. To examine the heterogeneity sources, we performed subgroup analyses based on sample size, patients’ origin, NOS score or follow-up time.

Furthermore, publication bias was explored through the evaluation of funnel plot asymmetry and Egger’s test. When Egger’s *p*-value < 0.05 and funnel plot was asymmetric, significant publication bias existed. In this case, trim-and-fill method was used to estimate a corrected effect size after adjustment, which helped determine whether the publication bias substantially affected the robustness of the pooled results.

## 3. Results

### 3.1. Study Characteristics

**Figure 1:**
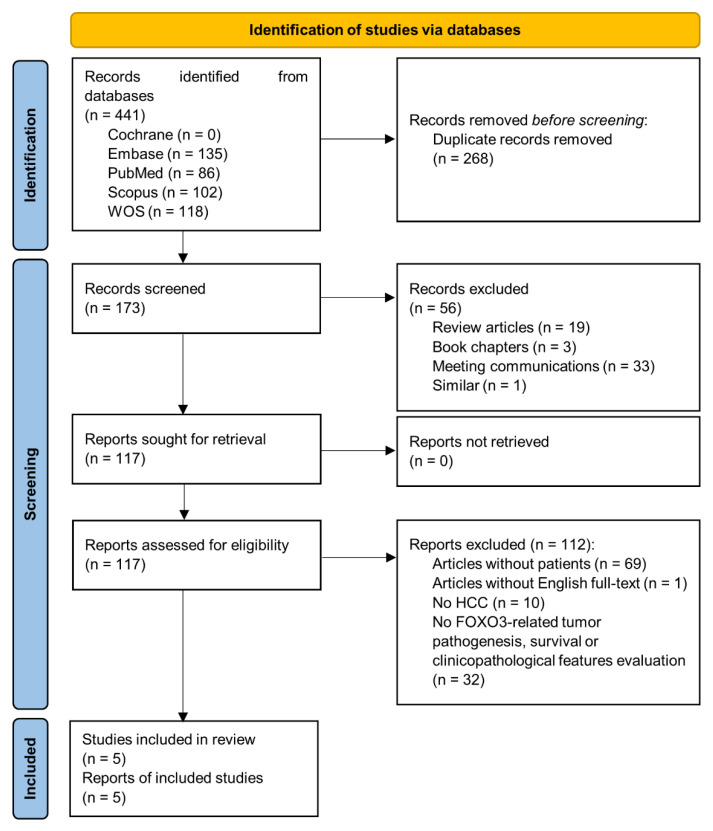
Preferred Reporting Items for Systematic Reviews and Meta-Analyses (PRISMA) flow diagram of study selection. FOXO3, forkhead box O3; HCC, hepatocellular carcinoma; WOS, Web of Science.

A total of 441 applicable records were identified through a database search, but 268 studies were duplicates, and after scanning titles and abstracts, another additional 56 non-original articles were excluded (reviews, book chapters, meeting communications or similar). The full-text of 117 articles was checked for eligibility, finding 69 articles without patients (only cell or animal models), one without English full-text, 10 without HCC patients and 32 without FOXO3-related tumor pathogenesis, survival or clinicopathological features evaluation. Therefore, these 112 papers were also removed from our study. Finally, five articles [12,15,16,19,20] were assessed for quality and data extraction. All these studies reached the quality threshold according to NOS score (Table 2) and were included for quantitative meta-analysis (Figure 1).

As reported in Table 2, the articles included in the present meta-analysis were published from 2009 to 2020. A total of 1059 HCC cases were included. Zhou et al. [15] did not directly report sample collection procedure or patient information. The rest of the samples were obtained by surgical resection, and the enrolled patients did not receive any pre- or post-operative treatment. The number of tumor samples across studies ranged from 91 to 365, and 537 of the totals (50.71%) exhibited FOXO3 overexpression. All included studies [12,15,16,19,20] provided data relating FOXO3 expression with OS, four articles [12,16,19,20] with clinicopathological features and two investigations [12,16] also compared FOXO3 levels between tumoral and healthy liver tissues. Only one study [16] evaluated disease-free survival (DFS). Therefore, the association of FOXO3 expression with DFS was impossible to analyze. 

Aside from Zhou et al. [15], which did not inform about the patients’ origin, all reported patients came from Asia, mainly from China (73.05%) [12,19,20] and the rest from Korea (26.95%) [16]. Furthermore, even though Ahn et al. [16] and Zhou et al. [15] did not provide gender data, the reported population was predominantly male (76.53%). Regarding risk factors, within the five papers included, three evaluated hepatitis B infection [16,19,20], and only one assessed hepatitis C and alcoholic condition [16]. All these studies were performed in HCC patients from Asia, and it should be mentioned that hepatitis B is the main etiological factor in this region [1]. Likewise, two researches [19,20] also validated the presence of cirrhosis in HCC patients. In summary, considering the total number of patients for whom the corresponding information is known, 73.28% of patients suffered from hepatitis B, 31.35% from hepatitis C, 32.64% from cirrhosis, 34.05% from alcoholism and 26.49% from hepatitis B, C and alcoholism simultaneously.

### 3.2. Association of FOXO3 Expression with HCC Pathogenesis

**Figure 2:**
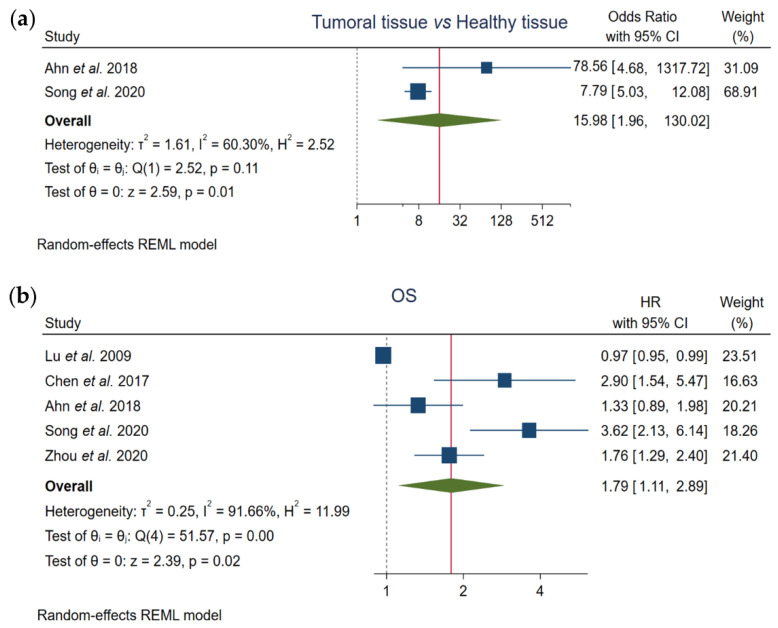
Forest plots of the studies assessing the relationship between forkhead box O3 (FOXO3) overexpression and (**a**) tumor pathogenesis or (**b**) overall survival (OS) in hepatocellular carcinoma (HCC) patients. CI, confidence interval; HR, hazard ratio; OS, overall survival; REML, Restricted Maximum Likelihood.

Two out of the five studies included in this meta-analysis compared FOXO3 levels between HCC samples and normal liver tissues. Pooled results showed that enhanced FOXO3 expression is significantly related to HCC development (OR, 15.98; 95% CI, 1.96–130.02; *p* = 0.01), finding an elevated heterogeneity (I^2^ = 60.30%, Q-test *p* = 0.11) (Figure 2a, Table 3).

### 3.3. Correlation between FOXO3 Expression and OS

Moreover, we assessed the prognostic value of FOXO3 expression using the total of the enrolled articles. Pooled results showed a significant correlation between high FOXO3 expression and OS (HR, 1.79; 95% CI, 1.11–2.89; *p* = 0.02), and a great heterogeneity across studies was detected (I^2^ = 91.66%, Q-test *p* = 0.00) (Figure 2b, Table 3). 

### 3.4. Correlation of FOXO3 Expression with Clinicopathological Features

**Figure 3:**
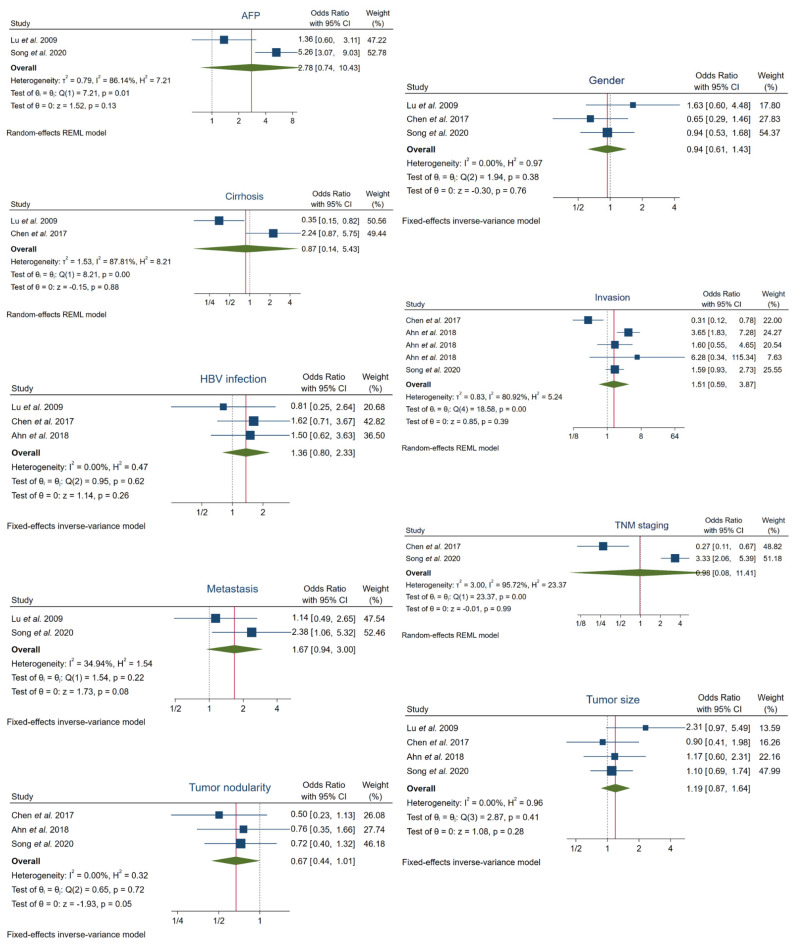
Forest plots of the studies assessing the relationship between FOXO3 overexpression and specific clinicopathological features in HCC patients. AFP, alpha-fetoprotein; CI, confidence interval; HBV, hepatitis B virus; REML, Restricted Maximum Likelihood; TNM, tumor-node-metastasis.

We pooled the available data from the selected five papers to determine the hypothetical correlation between high FOXO3 expression and several clinicopathological characteristics. However, enhanced FOXO3 expression was not statistically correlated with the evaluated features, which included: AFP (50 ng/mL) (OR, 2.78; 95% CI, 0.74–10.43; *p* = 0.13), cirrhosis (OR, 0.87; 95% CI, 0.14–5.43; *p* = 0.88), gender (OR, 0.94; 95% CI, 0.61–1.43; *p* = 0.76), hepatitis B virus (HBV) infection (OR, 1.36; 95% CI, 0.80–2.33; *p* = 0.26), invasion (OR, 1.51; 95% CI, 0.59–3.87; *p* = 0.39), metastasis (OR, 1.67; 95% CI, 0.94–3.00; *p* = 0.08), TNM staging (I–II, III–IV) (OR, 0.98; 95% CI, 0.08–11.41; *p* = 0.99), tumor nodularity (OR, 0.67; 95% CI, 0.44–1.01; *p* = 0.054) and tumor size (5 cm) (OR, 1.19; 95% CI, 0.87–1.64; *p* = 0.28) (Figure 3, Table 3).

Furthermore, heterogeneity tests revealed that heterogeneity was substantial in the following cases: AFP (I^2^ = 86.14%, Q-test *p* = 0.01), cirrhosis (I^2^ = 87.81%, Q-test *p* = 0.00), invasion (I^2^ = 80.92%, Q-test *p* = 0.00) and TNM staging (I^2^ = 95.72%, Q-test *p* = 0.00). Otherwise, assumable heterogeneity was found in gender (I^2^ = 0.00%, Q-test *p* = 0.38), HBV infection (I^2^ = 0.00%, Q-test *p* = 0.62), metastasis (I^2^ = 34.94%, Q-test *p* = 0.22), tumor nodularity (I^2^ = 0.00%, Q-test *p* = 0.72) and tumor size (I^2^ = 0.00%, Q-test *p* = 0.41) (Figure 3, Table 3).

### 3.5. Subgroup Analysis

Subgroup analysis for heterogeneous parameters was performed according to sample size, NOS score, patients’ origin or follow-up time, in order to examine the possible heterogeneity sources. It should be mentioned that subgroups composed of only one report were not considered.

**Figure 4:**
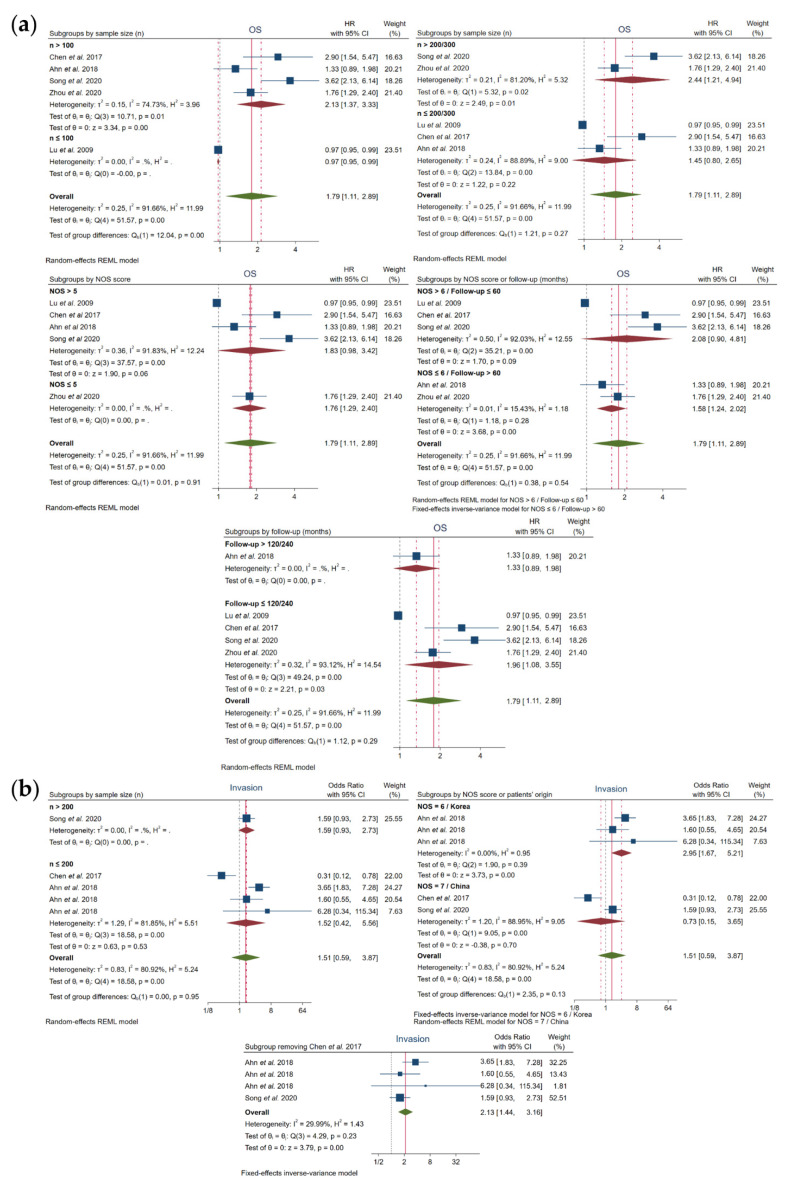
Forest plots after subgroup analysis of the studies assessing the relationship between FOXO3 overexpression and (**a**) OS or (**b**) invasion in HCC patients. CI, confidence interval; HR, hazard ratio; NOS, Newcastle–Ottawa scale; OS, overall survival; REML, Restricted Maximum Likelihood.

In regard to OS, a correlation was shown with high FOXO3 expression when subgroups were based on sample size n > 100 (HR, 2.13; 95% CI, 1.37–3.33; *p* = 0.00) and n > 200/300 (HR, 2.44; 95% CI, 1.21–4.94; *p* = 0.01), as well as on follow-up months (≤120/240) (HR, 1.96; 95% CI, 1.08–3.55; *p* = 0.03). However, heterogeneity still continued to be high in all of these previous subgroups: sample size n > 100 (I^2^ = 74.73% and Q-test *p* = 0.01), n > 200/300 (I^2^ = 81.20% and Q-test *p* = 0.02) and follow-up months (≤120/240) (I^2^ = 93.12% and Q-test *p* = 0.00) (Figure 4a, Table 4). 

Otherwise, FOXO3 overexpression was significantly associated to OS when NOS ≤ 6 or follow-up >60 months (HR, 1.58; 95% CI, 1.24–2.02; *p* = 0.00), and heterogeneity was successfully solved, as shown by I^2^ = 15.43% and Q-test *p* = 0.28 (Figure 4a, Table 4).

Additionally, heterogeneity in invasion was resolved based on NOS = 6 and Korean provenance (I^2^ = 0.00% and Q-test *p* = 0.39), showing also a strong association with high levels of FOXO3 (OR, 2.95; 95% CI, 1.67–5.21; *p* = 0.00). Curiously, the elimination of Chen et al. [19] also led to an assumable heterogeneity (I^2^=29.99% and Q-test *p* = 0.23) and a significant relation with FOXO3 overexpression (OR, 2.13; 95% CI, 1.44–3.16; *p* = 0.00) (Figure 4b, Table 4). 

The rest of the subgroups exhibited an elevated heterogeneity and did not follow a correlation with FOXO3 expression (Figure 4, Table 4). However, overall, subgroup analysis helped disclose that sample size, follow-up time, NOS score and patients’ origin contribute, at least in part, to the reported heterogeneity.

### 3.6. Publication Bias

**Figure 5:**
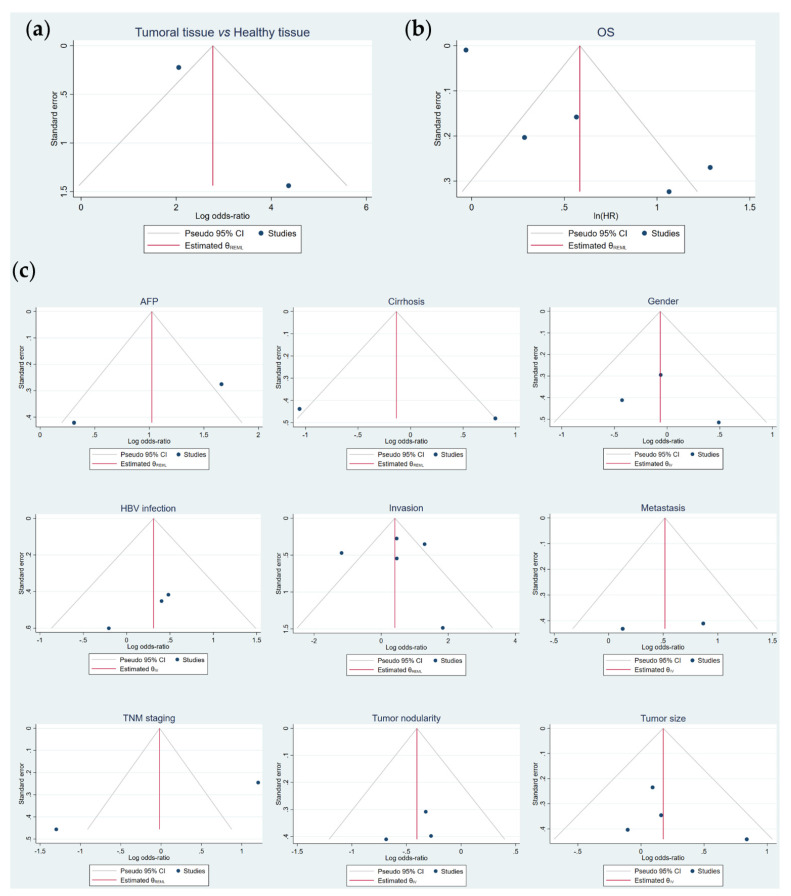
Publication bias evaluation of the potential correlation of FOXO3 overexpression with (**a**) tumor pathogenesis, (**b**) OS and (**c**) the assessed clinicopathological features by funnel plots asymmetry. AFP, alpha-fetoprotein; CI, confidence interval; HBV, hepatitis B virus; HR, hazard ratio; IV, Inverse Variance; OS, overall survival; REML, Restricted Maximum Likelihood; TNM, tumor-node-metastasis.

Although an Egger’s test could not be accomplished for the comparison of HCC tumor vs. non-tumor samples (Table 5), funnel plot analysis likely showed a slight asymmetry (Figure 5a). In regard to OS, asymmetry was observed (Figure 5b) and a significant result was obtained in the Egger’s test (*p* = 0.00) (Table 5), which denoted publication bias. Hence, the trim-and-fill method was performed, but no “missing” studies were imputed, and the global effect size remained unchanged (Table 5). Conversely, publication bias was not detected for all the assessed clinicopathological characteristics (Figure 5c, Table 5).

## 4. Discussion

Primary liver cancer, mainly represented by HCC [2,6,25], remains a global unsolved health challenge [1,2]. Asymptomatic presentation at early stages, deficient diagnostic methodology and post-therapy recurrence are common features of this lethal tumor with a remarkably poor prognosis [2,6,15,16,19,25]. Moreover, although increasing efforts are being put into HCC biomarker discovery [24,25,26,27], effective molecules that assist tumor detection and predict therapy response are still lacking.

FOXO3, an important member belonging to the evolutionary conserved FOXO sub-family [7,8,10], is a central transcription factor that governs downstream targets involved in key cellular processes [10]. It has been reported that the deregulation of FOXO3 expression is involved in cancer emergence [10,12,16] and progression [12,16,19]. FOXO3, which is mainly expressed by liver cells [7], seems to be connected to the development of hepatic disease [12], but the exact linkage between FOXO3 expression and primary liver cancer has not been uncovered yet.

Therefore, we performed the present systematic review with meta-analysis to accurately determine the association of FOXO3 overexpression with tumor development, survival outcome and clinicopathological factors, examining the potential usefulness of this factor as a diagnostic and prognostic biomarker for HCC monitoring.

A total of five high-quality studies, enrolling 1059 HCC cases, were selected for conducting this meta-analysis. Approximately half of the cases included in this investigation showed high FOXO3 expression, and most patients came from Asia, mainly from China, which is not surprising as this geographical area accounts for nearly 75% of HCC incidence worldwide [6] and about 50% of new HCC patients are Chinese [28]. 

The current meta-analysis revealed a significant correlation between FOXO3 high expression and HCC pathogenesis. Interestingly, Lu et al. [29] evidenced that FOXO3 is highly expressed and overactivated in HCC patients, which was associated with strong liver damage and overexpression of HCC-related genes, suggesting that FOXO3 overexpression is involved in tumorigenesis promotion. Additional reports from pre-clinical studies also indicated that FOXO3 upregulation is related to HCC oncogenicity via the overexpression of the long noncoding RNAs (lncRNAs) PRR34 antisense RNA 1 (PRR34-AS1) [17] and LOC554202 [30], or by the circular RNA circFBXO11/miR-605 axis [13]. Contrary to all the above-mentioned results, the article by Wu et al. [31] described that reduction in FOXO3 nuclear translocation and activity could be involved in sepiapterin reductase-mediated HCC progression. Considering all the previous evidence, which mostly defends a tumor-promoting action of the positive regulation of FOXO3, our meta-analysis certainly supports the findings reported by the majority of studies and underscores the role of FOXO3 overexpression on fostering HCC development. Hence, the upregulation of FOXO3 may constitute a suitable diagnostic factor able to complement classic techniques.

Meanwhile, primary pooled results showed a significant association between FOXO3 overexpression and shorter OS of HCC patients. Chen et al. [32] emphasized that FOXO3 expression is related to shorter survival time and cancer progression in invasive ductal breast carcinoma, which was also observed in glioblastoma human samples [33]. Likewise, high levels of this factor were significantly related to a shorter DFS time and an increased Ki-67 proliferation index in triple-negative breast cancer (TNBC) patients [34]. Otherwise, Zhao et al. [35] observed that FOXO3 transactivity is impaired in HCC due to FOXO3 downregulation, which could be linked with the enhancement of cell proliferation promoted by thyroid hormone receptor-interacting protein 6 (TRIP6). However, correlation between TRIP6 and FOXO3 expression in HCC individuals and its impact on survival rate were not assessed [35]. Therefore, there is no conclusive statement about the impact of FOXO3 on HCC prognosis based on such a study.

Considering FOXO3 as a potential biomarker in HCC constitutes a novel approach. As a consequence, there is still little research in the literature that analyzes the possible connection between FOXO3 levels and survival outcome, being all this evidence included in the current systematic review with meta-analysis. Even though our study proved the potential of FOXO3 as a negative prognostic factor in HCC, additional large-scale investigations should be performed in this cancer type to confirm such an encouraging result.

Regarding the evaluated clinicopathological features, subgroup analysis showed that invasion statistically correlates with high levels of FOXO3 in articles with a medium–high NOS score or those harboring Korean patients, suggesting that elevated FOXO3 expression may trigger HCC invasiveness. Additionally, it has been reported that FOXO3 expression accentuates invasiveness and tumor expansion in glioblastoma [33], pancreatic cancer [36] and HeLa and melanoma MDA-MB-435 cells [37], being also correlated to perineural invasion in TNBC samples [34].

Contrariwise, FOXO3 oppositely impacted the invasive capabilities of breast tumors, depending on the estrogen receptor α (ERα) status [38]. Besides, Yang et al. [39] demonstrated the potential of bortezomib to suppress cell migration and invasion via FOXO3 upregulation in cholangiocarcinoma and HCC in vitro models. However, these results were not tested in vivo nor in HCC patients. Moreover, it needs to be mentioned that bortezomib is not currently approved for HCC treatment, due to it not showing enough efficacy against this tumor, and it does not represent a major chemotherapeutic drug within the HCC field. It is known that FOXO3 displays a dual role in cancer, promoting malignant phenotypes or inhibiting cancer progression depending on the tumor type or specific tumor-related circumstances. This fact likely drives contrasting findings among different reports. Therefore, although our meta-analysis revealed for the first time a significant correlation between enhanced FOXO3 levels and the risk of invasion, further studies are required to unequivocally unravel the impact of FOXO3 on the modulation of HCC invasion capability.

In regard to clinicopathological factors evaluated other than invasion, there was not a significant association between FOXO3 overexpression and AFP levels, cirrhosis, gender, HBV infection, metastasis, TNM staging, tumor nodularity or tumor size. However, despite no correlation observed between FOXO3 levels and HBV infection, Chen et al. [40] proved that FOXO3 participates in the HBV-mediated HCC tumorigenesis. Given the absence of additional HCC investigations, novel studies analyzing the relationship of HBV infection or other clinicopathological factors with FOXO3 expression will help unravel the potential of this central transcription factor to predict HCC-associated features and reinforce its usefulness as a novel biomarker.

With respect to the possible association between FOXO3 expression and the aforementioned clinicopathological parameters in other tumors, an investigation conducted with nasopharyngeal carcinoma samples observed that low FOXO3 expression correlates with advanced clinical stages and higher T stages, apart from lymph node metastasis and distant metastasis [41]. Reduced FOXO3 levels in colorectal cancer [42], esophageal squamous cell carcinoma (ESCC) [43] and pancreatic ductal adenocarcinoma samples [44] also correlated with more advanced disease. Meanwhile, deregulation of FOXO3 levels has shown to differentially influence lymph node metastasis in invasive ductal carcinoma [32], TNBC [34] and bladder carcinoma [45], finding that the interplay β-catenin-FOXO3 can also behave as a metastasis promoter in colon cancer [46]. Moreover, FOXO3 downregulation in ESCC patients accounted for lymph node metastasis [43], and its low expression correlated with a larger tumor size in gastric adenocarcinoma [47]. 

Collectively, all the available evidence concerning FOXO3 regulation and the associated cancer features highlights the double-edged action played by this crucial transcription factor, finding that deregulation of FOXO3 expression and activity may definitely determine tumor promotion or suppression depending on the cancer type, cellular context or genomic profile.

In summary, our study is the first comprehensive, detailed and systematic meta-analysis evaluating and demonstrating the diagnostic and prognostic value of FOXO3 in HCC, thus covering an unexplored research pathway. It is worth mentioning that all published and available clinical evidence on the association of FOXO3 with HCC development, survival parameters and clinicopathological features was checked and, if appropriated, included in this work. Moreover, our meta-analysis contains important and informative analysis, such as heterogeneity assessment, subgroup analysis and publication bias evaluation. Therefore, although further studies are needed to corroborate our findings, this novel systematic review with meta-analysis constitutes the first addressing the clinical significance of FOXO3 as a biomarker in HCC, thereby setting the basis for future investigations and providing a new potential molecule that could successfully assist HCC detection and prognostic evaluation.

However, there are still some limitations in the current study that need to be considered. The number of articles included both in qualitative and quantitative synthesis was low, and the evaluation of the diagnostic and clinicopathological significance of FOXO3 in HCC involved even fewer reports, due to these data being missing in some of the studies. Thus, the volume of the research was relatively small, and more investigations are required to verify and complete the results obtained by this innovative meta-analysis. 

On the other hand, although only one paper was excluded during the assessment of eligible articles because the full-text was not written in English, exclusion of studies published in languages other than English probably accounts for publication bias, discarding investigations with relevant results. Despite one of the five included articles not reporting the patients’ ethnicity, the rest of the studies were carried out with Asian patient samples. There is a lack of investigations performed with people from other geographical regions, which leads to a disbalance in terms of patients’ origin, likely contributing to bias apparition. Additionally, not every included report evaluated HCC-associated risk factors, such as HBV infection, thereby missing relevant information that could condition global results. In regard to studies evaluating FOXO3 levels by IHC, they established very similar but not completely identical criteria for the definition of “high” FOXO3 expression, which could cause mild heterogeneity. HRs from two out of the five included studies had to be estimated from survival curves, as this information was not directly reported, which could slightly contribute to variability apparition among articles. Furthermore, only one of the selected articles provided DFS information, an interesting prognosis-related variable, but this fact prevented determining its potential association with FOXO3 expression.

## 5. Conclusions

In conclusion, our systematic review with meta-analysis has been the first to evaluate the potential of the transcription factor FOXO3 as a novel and functional biomarker in cancer and, more precisely, in HCC. This novel study demonstrated that an increased expression of FOXO3 may be an unfavorable clinical factor with diagnostic and prognostic value in HCC, being related to tumor development, poor OS and a high probability of invasion. Thus, the evaluation of FOXO3 levels constitutes a promising strategy to optimize and complement HCC detection and, specifically, to guide patient surveillance and make an accurate prognosis. However, these findings need to be confirmed by additional high-quality, well-designed and large-scale investigations.

## Figures and Tables

**Table 1:** Search strategy for each database (until 30th April 2021 included).

Database	Search Strategy
Cochrane Library	(foxo3 OR foxo3a OR fkhrl1 OR Forkhead box protein O3 OR Forkhead box O3 OR forkhead box O-3 OR AF6q21 OR Forkhead in rhabdomyosarcoma-like 1 OR fkhrl1p2 OR foxo2a) AND (hepatocellular carcinoma OR hepatocarcinoma OR HCC): ti, ab, kw
Embase	(‘foxo3’ OR ‘foxo3a’ OR ‘fkhrl1′ OR ‘forkhead box protein o3′ OR ‘forkhead box o3’ OR ‘forkhead box o-3’ OR ‘af6q21’ OR ‘forkhead in rhabdomyosarcoma-like 1′ OR ‘fkhrl1p2’ OR ‘foxo2a’) AND (‘hepatocellular carcinoma’ OR ‘hepatocarcinoma’ OR ‘hcc’)
PubMed	(“foxo3”[All Fields] OR “foxo3a”[All Fields] OR “fkhrl1”[All Fields] OR “Forkhead box protein O3”[All Fields] OR “Forkhead box O3”[All Fields] OR “forkhead box O-3”[All Fields] OR “AF6q21”[All Fields] OR “Forkhead in rhabdomyosarcoma-like 1”[All Fields] OR “fkhrl1p2”[All Fields] OR “foxo2a”[All Fields]) AND (“hepatocellular carcinoma”[All Fields] OR “hepatocarcinoma”[All Fields] OR “HCC”[All Fields])
Scopus	TITLE-ABS-KEY ((“foxo3” OR “foxo3a” OR “fkhrl1” OR “Forkhead box protein O3” OR “Forkhead box O3” OR “forkhead box O-3” OR “AF6q21” OR “Forkhead in rhabdomyosarcoma-like 1” OR “fkhrl1p2” OR “foxo2a”) AND (“hepatocellular carcinoma” OR “hepatocarcinoma” OR “HCC”))
WOS Core Collection	TS = ((“foxo3” OR “foxo3a” OR “fkhrl1” OR “Forkhead box protein O3” OR “Forkhead box O3” OR “forkhead box O-3” OR “AF6q21” OR “Forkhead in rhabdomyosarcoma-like 1” OR “fkhrl1p2” OR “foxo2a”) AND (“hepatocellular carcinoma” OR “hepatocarcinoma” OR “HCC”)) *Indexes = SCI-EXPANDED, SSCI, A&HCI, CPCI-S, CPCI-SSH, BKCI-S, BKCI-SSH, ESCI, CCR-EXPANDED, IC Timespan = All years*

WOS, Web of Science.

**Table 2:** Baseline characteristics of selected articles.

Study	Lu et al. [20]	Chen et al. [19]	Ahn et al. [16]	Song et al. [12]	Zhou et al. [15]
Year	2009	2017	2018	2020	2020
Tumor sample size	91 (74) *	102	187	314	365
Tumor sample size (M/F)	71/20 (61/13) *	62/40	NR	255/59	NR
Patients’ origin	China	China	Korea	China	NR
Intervention	Surgical resection	Surgical resection	Surgical resection	Surgical resection	NR
Pre- or post-surgery treatment	None	None	NR	NR	NR
Age range	32–72	NR	NR	NR	NR
Mean/median age	51.37 ± 10.50	NR	NR	NR	NR
Study quality	7/9	7/9	6/9	7/9	5/9
FOXO3 levels measurement	IHC	IHC	IHC	IHC	RNA-Seq **
Survival analysis	CS	OS	OS/DFS	OS	OS
HR	Reported	Reported	Reported	Estimated	Estimated
Healthy liver sample size	NR	NR	21	150	NR
Definition of “high” FOXO3 expression	>0.40	>4 ^1^	≥1 ^2^	≥3 ^3^	NR
Number of tumor samples with “high” FOXO3 expression	45 (37) *	42	121	238	91
Number of healthy liver samples with “high” FOXO3 expression	NR	NR	0	43	NR

CS, cumulative survival; DFS, disease-free survival; F, female; FOXO3, forkhead box O3; HR, hazard ratio; IHC, immunohistochemistry; M, male; NR, not reported; OS, overall survival. * Tumor samples included for survival analysis. ** From UALCAN database. ^1^ Final scores were calculated by multiplying the score obtained for percentage of positive cells (0, ≤5%; 1, 5–25%; 2, 26–50%; 3, 51–75%; 4, >75%) by the score registered for staining intensity (0, no signal; 1, weak; 2, moderate; 3, strong). ^2^ Final scores were calculated by multiplying the score obtained for percentage of positively stained tumor cells (0, 0–10%; 1, 11–25%; 2, 26–50%; 3, 51–75%; 4, 76–100%) by the score registered for staining intensity (0, negative; 1, weak; 2, intermediate; 3, strong). ^3^ Final scores were calculated by multiplying the score obtained for percentage of positive cells stained (0, no staining; 1, 1 ≤ 1 < 25%; 2, 25% ≤ 2 < 50%; 3, 50% ≤ 3 < 75%; 4, ≥75%) by the score registered for staining intensity (0, negative; 1, weak; 2, moderate; 3, strong).

**Table 3:** Analysis of the association of FOXO3 overexpression with tumor development, survival and clinicopathological factors.

Parameter	Number of Studies (n)	Number of Cases (n)	Samples with High FOXO3 Expression (n)	High FOXO3 Expression (%)	Pooled OR or HR		Test for Heterogeneity		Model Used
					95% CI	*p*-Value	I^2^	Q-Test *p*-Value	
HCC pathogenesis									
Tumoral tissue vs. Healthy tissue	2	672	402	59.82%	15.98 (1.96–130.02)	0.01	60.30%	0.11	REM
OS	5	1042	529	50.77%	1.79 (1.11–2.89)	0.02	91.66%	0.00	REM
Clinicopathological features									
AFP	2	346	153	44.22%	2.78 (0.74–10.43)	0.13	86.14%	0.01	REM
Cirrhosis	2	193	87	45.08%	0.87 (0.14–5.43)	0.88	87.81%	0.00	REM
Gender	3	507	211	41.62%	0.94 (0.61–1.43)	0.76	0.00%	0.38	FEM
HBV infection	3	378	207	54.76%	1.36 (0.80–2.33)	0.26	0.00%	0.62	FEM
Invasion	5	890	497	55.84%	1.51 (0.59–3.87)	0.39	80.92%	0.00	REM
Metastasis	2	400	168	42.00%	1.67 (0.94–3.00)	0.08	34.94%	0.22	FEM
TNM staging	2	414	166	40.10%	0.98 (0.08–11.41)	0.99	95.72%	0.00	REM
Tumor nodularity	3	603	287	47.60%	0.67 (0.44–1.01)	0.054	0.00%	0.72	FEM
Tumor size	4	687	318	46.29%	1.19 (0.87–1.64)	0.28	0.00%	0.41	FEM

AFP, alpha-fetoprotein; CI, confidence interval; FEM, fixed-effects model; FOXO3, forkhead box O3; HBV, hepatitis B virus; HCC, hepatocellular carcinoma; HR, hazard ratio; OR, odds ratio; OS, overall survival; REM, random-effects model; TNM, tumor-node-metastasis.

**Table 4:** Subgroup analysis.

Subgroups	Number of Studies (n)	Number of Cases (n)	Samples with High FOXO3 Expression (n)	High FOXO3 Expression (%)	Pooled OR or HR		Test for Heterogeneity		Model Used
					95% CI	*p*-Value	I^2^	Q-Test *p*-Value	
OS									
Sample size (n)									
*n* > 100	4	968	492	50.83%	2.13 (1.37–3.33)	0.00	74.73%	0.01	REM
*n* ≤ 100	1	74	37	50.00%	0.97 (0.95–0.99)	-	-	-	-
*n* > 200/300	2	679	329	48.45%	2.44 (1.21–4.94)	0.01	81.20%	0.02	REM
*n* ≤ 200/300	3	363	200	55.10%	1.45 (0.80–2.65)	0.22	88.89%	0.00	REM
NOS score (threshold 5)									
NOS > 5	4	677	438	64.70%	1.83 (0.98–3.42)	0.06	91.83%	0.00	REM
NOS ≤ 5	1	365	91	24.93%	1.76 (1.29–2.40)	-	-	-	-
NOS score (threshold 6)									
NOS > 6	3	490	317	64.69%	2.08 (0.90–4.81)	0.09	92.03%	0.00	REM
NOS ≤ 6	2	552	212	38.41%	1.58 (1.24–2.02)	0.00	15.43%	0.28	FEM
Follow-up (months)									
>60	2	552	212	38.41%	1.58 (1.24–2.02)	0.00	15.43%	0.28	FEM
≤60	3	490	317	64.69%	2.08 (0.90–4.81)	0.09	92.03%	0.00	REM
>120/240	1	187	121	64.71%	1.33 (0.89–1.98)	-	-	-	-
≤120/240	4	855	408	47.72%	1.96 (1.08–3.55)	0.03	93.12%	0.00	REM
Invasion									
Sample size (n)									
*n* > 200	1	227	92	40.53%	1.59 (0.93–2.73)	-	-	-	-
*n* ≤ 200	4	663	405	61.09%	1.52 (0.42–5.56)	0.53	81.85%	0.00	REM
NOS score									
NOS = 6	3	561	363	64.71%	2.95 (1.67–5.21)	0.00	0.00%	0.39	FEM
NOS = 7	2	329	134	40.73%	0.73 (0.15–3.65)	0.70	88.95%	0.00	REM
Patients’ origin									
China	2	329	134	40.73%	0.73 (0.15–3.65)	0.70	88.95%	0.00	REM
Korea	3	561	363	64.71%	2.95 (1.67–5.21)	0.00	0.00%	0.39	FEM
Without Chen et al. [19]									
	4	788	455	57.74%	2.13 (1.44–3.16)	0.00	29.99%	0.23	FEM

CI, confidence interval; FEM, fixed-effects model; FOXO3, forkhead box O3; HR, hazard ratio; NOS, Newcastle–Ottawa scale; OR, odds ratio; OS, overall survival; REM, random-effects model.

**Table 5:** Evaluation of publication bias.

Parameter	Number of Studies (n)	Egger’s Test *p*-Value	Model Used	Trim-and-Fill OR or HR (95% CI)	Imputed Studies(n)
HCC pathogenesis					
Tumoral tissue vs. Healthy tissue	2	*	REM	-	-
OS	5	0.00	REM	1.79 (1.11–2.89)	0
Clinicopathological features					
AFP	2	*	REM	-	-
Cirrhosis	2	*	REM	-	-
Gender	3	0.59	FEM	-	-
HBV infection	3	0.33	FEM	-	-
Invasion	5	0.57	REM	-	-
Metastasis	2	0.22	FEM	-	-
TNM staging	2	*	REM	-	-
Tumor nodularity	3	0.67	FEM	-	-
Tumor size	4	0.43	FEM	-	-

AFP, alpha-fetoprotein; CI, confidence interval; FEM, fixed-effects model; HBV, hepatitis B virus; HCC, hepatocellular carcinoma; HR, hazard ratio; OR, odds ratio; OS, overall survival; REM, random-effects model; TNM, tumor-node-metastasis. * Convergence not achieved during tau2 estimation.

## Data Availability

The data presented in this study are available in this article.

## Supplementary Materials

The following are available online at https://www.mdpi.com/article/10.3390/cancers13215349/s1—Table S1: PRISMA 2020 checklist; Table S2: PRISMA 2020 for Abstracts checklist; Table S3: antibodies and procedure of staining employed by those included articles that analyzed FOXO3 expression using IHC.

## References

[1] Sung H., Ferlay J., Siegel R.L., Laversanne M., Soerjomataram I., Jemal A., Bray F. (2021). Global cancer statistics 2020: GLOBOCAN estimates of incidence and mortality worldwide for 36 cancers in 185 countries. CA Cancer J. Clin..

[2] Llovet J.M., Kelley R.K., Villanueva A., Singal A.G., Pikarsky E., Roayaie S., Lencioni R., Koike K., Zucman-Rossi J., Finn R.S. (2021). Hepatocellular carcinoma. Nat. Rev. Dis. Primers.

[3] Forner A., Reig M., Bruix J. (2018). Hepatocellular carcinoma. Lancet.

[4] Kulik L., El-Serag H.B. (2019). Epidemiology and management of hepatocellular carcinoma. Gastroenterology.

[5] Petrick J.L., Florio A.A., Znaor A., Ruggieri D., Laversanne M., Alvarez C.S., Ferlay J., Valery P.C., Bray F., McGlynn K.A. (2020). International trends in hepatocellular carcinoma incidence, 1978–2012. Int. J. Cancer.

[6] Firkins J.L., Tarter R., Driessnack M., Hansen L. (2021). A closer look at quality of life in the hepatocellular carcinoma literature. Qual. Life Res..

[7] Calissi G., Lam E.W.-F., Link W. (2021). Therapeutic strategies targeting FOXO transcription factors. Nat. Rev. Drug Discov..

[8] Coomans De Brachène A., Demoulin J.-B. (2016). FOXO transcription factors in cancer development and therapy. Cell. Mol. Life Sci..

[9] Carbajo-Pescador S., Mauriz J.L., García-Palomo A., González-Gallego J. (2014). FoxO proteins: Regulation and molecular targets in liver cancer. Curr. Med. Chem..

[10] Liu Y., Ao X., Ding W., Ponnusamy M., Wu W., Hao X., Yu W., Wang Y., Li P., Wang J. (2018). Critical role of FOXO3a in carcinogenesis. Mol. Cancer.

[11] Carbajo-Pescador S., Steinmetz C., Kashyap A., Lorenz S., Mauriz J.L., Heise M., Galle P.R., González-Gallego J., Strand S. (2013). Melatonin induces transcriptional regulation of Bim by FoxO3a in HepG2 cells. Br. J. Cancer.

[12] Song S.-S., Ying J.-F., Zhang Y.-N., Pan H.-Y., He X.-L., Hu Z.-M., Wang H.-J., Dou X.-B., Mou X.-Z. (2020). High expression of FOXO3 is associated with poor prognosis in patients with hepatocellular carcinoma. Oncol. Lett..

[13] Li J., Qin X., Wu R., Wan L., Zhang L., Liu R. (2020). Circular RNA circFBXO11 modulates hepatocellular carcinoma progress and oxaliplatin resistance through miR-605/FOXO3/ABCB1 axis. J. Cell. Mol. Med..

[14] Liang C., Dong Z., Cai X., Shen J., Xu Y., Zhang M., Li H., Yu W., Chen W. (2020). Hypoxia induces sorafenib resistance mediated by autophagy via activating FOXO3a in hepatocellular carcinoma. Cell Death Dis..

[15] Zhou Q., Li Z., Song L., Mu D., Wang J., Tian L., Liao Y. (2020). Whole-exome mutational landscape of metastasis in patient-derived hepatocellular carcinoma cells. Genes Dis..

[16] Ahn H., Kim H., Abdul R., Kim Y., Sim J., Choi D., Paik S.S., Shin S.-J., Kim D.-H., Jang K. (2018). Overexpression of forkhead box O3a and its association with aggressive phenotypes and poor prognosis in human hepatocellular carcinoma. Am. J. Clin. Pathol..

[17] Yang S., Pang L., Dai W., Wu S., Ren T., Duan Y., Zheng Y., Bi S., Zhang X., Kong J. (2021). Role of forkhead box O proteins in hepatocellular carcinoma biology and progression (Review). Front. Oncol..

[18] Lin Z., Niu Y., Wan A., Chen D., Liang H., Chen X., Sun L., Zhan S., Chen L., Cheng C. (2020). RNA m^6^A methylation regulates sorafenib resistance in liver cancer through FOXO3-mediated autophagy. EMBO J..

[19] Chen Y., Wang J., Zhang L., Yuan P., Lei L., Liu D. (2017). Decreased expression of forkhead box O3 in human hepatocellular carcinoma and its prognostic significance. Int. J. Clin. Exp. Med..

[20] Lu M., Ma J., Xue W., Cheng C., Wang Y., Zhao Y., Ke Q., Liu H., Liu Y., Li P. (2009). The expression and prognosis of FOXO3a and Skp2 in human hepatocellular carcinoma. Pathol. Oncol. Res..

[21] Page M.J., McKenzie J.E., Bossuyt P.M., Boutron I., Hoffmann T.C., Mulrow C.D., Shamseer L., Tetzlaff J.M., Akl E.A., Brennan S.E. (2021). The PRISMA 2020 statement: An updated guideline for reporting systematic reviews. BMJ.

[22] The Newcastle-Ottawa Scale (NOS) for Assessing the Quality of Nonrandomised Studies in Meta-Analyses. http://www.ohri.ca/programs/clinical_epidemiology/oxford.asp.

[23] Parmar M.K.B., Torri V., Stewart L. (1998). Extracting summary statistics to perform meta-analyses of the published literature for survival endpoints. Stat. Med..

[24] Méndez-Blanco C., Fernández-Palanca P., Fondevila F., González-Gallego J., Mauriz J.L. (2021). Prognostic and clinicopathological significance of hypoxia-inducible factors 1α and 2α in hepatocellular carcinoma: A systematic review with meta-analysis. Ther. Adv. Med. Oncol..

[25] Mansouri V., Razzaghi M., Nikzamir A., Ahmadzadeh A., Iranshahi M., Haghazali M., Hamdieh M. (2020). Assessment of liver cancer biomarkers. Gastroenterol. Hepatol. Bed Bench.

[26] Sukowati C.H.C., Cabral L.K.D., Tiribelli C., Pascut D. (2021). Circulating long and circular noncoding RNA as non-invasive diagnostic tools of hepatocellular carcinoma. Biomedicines.

[27] Pratama M.Y., Visintin A., Crocè L.S., Tiribelli C., Pascut D. (2020). Circulatory miRNA as a biomarker for therapy response and disease-free survival in hepatocellular carcinoma. Cancers.

[28] Gingold J.A., Zhu D., Lee D.-F., Kaseb A., Chen J. (2018). Genomic profiling and metabolic homeostasis in primary liver cancers. Trends Mol. Med..

[29] Lu M., Hartmann D., Braren R., Gupta A., Wang B., Wang Y., Mogler C., Cheng Z., Wirth T., Friess H. (2019). Oncogenic Akt-FOXO3 loop favors tumor-promoting modes and enhances oxidative damage-associated hepatocellular carcinogenesis. BMC Cancer.

[30] Yang L., Deng W., Zhao B., Xu Y., Wang X., Fang Y., Xiao H. (2021). FOXO3-induced lncRNA LOC554202 contributes to hepatocellular carcinoma progression via the miR-485-5p/BSG axis. Cancer Gene Ther..

[31] Wu Y., Du H., Zhan M., Wang H., Chen P., Du D., Liu X., Huang X., Ma P., Peng D. (2020). Sepiapterin reductase promotes hepatocellular carcinoma progression via FoxO3a/Bim signaling in a nonenzymatic manner. Cell Death Dis..

[32] Chen J., Gomes A.R., Monteiro L.J., Wong S.Y., Wu L.H., Ng T.T., Karadedou C.T., Millour J., Ip Y.-C., Cheung Y.N. (2010). Constitutively nuclear FOXO3a localization predicts poor survival and promotes Akt phosphorylation in breast cancer. PLoS ONE.

[33] Qian Z., Ren L., Wu D., Yang X., Zhou Z., Nie Q., Jiang G., Xue S., Weng W., Qiu Y. (2017). Overexpression of FoxO3a is associated with glioblastoma progression and predicts poor patient prognosis. Int. J. Cancer.

[34] Rehman A., Kim Y., Kim H., Sim J., Ahn H., Chung M.S., Shin S.-J., Jang K. (2018). FOXO3a expression is associated with lymph node metastasis and poor disease-free survival in triple-negative breast cancer. J. Clin. Pathol..

[35] Zhao W., Dai Y., Dai T., Xie T., Su X., Li J., Zhou X., Meng K., Zhao X. (2017). TRIP6 promotes cell proliferation in hepatocellular carcinoma via suppression of FOXO3a. Biochem. Biophys. Res. Commun..

[36] Yu S., Yu Y., Zhang W., Yuan W., Zhao N., Li Q., Cui Y., Wang Y., Li W., Sun Y. (2016). FOXO3a promotes gastric cancer cell migration and invasion through the induction of cathepsin L. Oncotarget.

[37] Storz P., Döppler H., Copland J.A., Simpson K.J., Toker A. (2009). FOXO3a promotes tumor cell invasion through the induction of matrix metalloproteinases. Mol. Cell. Biol..

[38] Sisci D., Maris P., Cesario M.G., Anselmo W., Coroniti R., Trombino G.E., Romeo F., Ferraro A., Lanzino M., Aquila S. (2013). The estrogen receptor α is the key regulator of the bifunctional role of FoxO3a transcription factor in breast cancer motility and invasiveness. Cell Cycle.

[39] Yang Z., Liu S., Zhu M., Zhang H., Wang J., Xu Q., Lin K., Zhou X., Tao M., Li C. (2016). PS341 inhibits hepatocellular and colorectal cancer cells through the FOXO3/CTNNB1 signaling pathway. Sci. Rep..

[40] Chen W., Jiang J., Gong L., Shu Z., Xiang D., Zhang X., Bi K., Diao H. (2021). Hepatitis B virus P protein initiates glycolytic bypass in HBV-related hepatocellular carcinoma via a FOXO3/miRNA-30b-5p/MINPP1 axis. J. Exp. Clin. Cancer Res..

[41] Shou Z., Lin L., Liang J., Li J.-L., Chen H.-Y. (2012). Expression and prognosis of FOXO3a and HIF-1α in nasopharyngeal carcinoma. J. Cancer Res. Clin. Oncol..

[42] Bullock M.D., Bruce A., Sreekumar R., Curtis N., Cheung T., Reading I., Primrose J.N., Ottensmeier C., Packham G.K., Thomas G. (2013). FOXO3 expression during colorectal cancer progression: Biomarker potential reflects a tumour suppressor role. Br. J. Cancer.

[43] Lu Y., Yu J., Yang Z., Zhu G., Gao P., Wang H., Chen S., Zhang J., Liu M., Niu Y. (2018). Promoter hypomethylation mediated upregulation of MicroRNA-10b-3p targets FOXO3 to promote the progression of esophageal squamous cell carcinoma (ESCC). J. Exp. Clin. Cancer Res..

[44] Luo X., Yang Z., Liu X., Liu Z., Miao X., Li D., Zou Q., Yuan Y. (2017). The clinicopathological significance of forkhead box P1 and forkhead box O3a in pancreatic ductal adenocarcinomas. Tumor Biol..

[45] Wang Y., Kang X.-L., Zeng F.-C., Xu C.-J., Zhou J.-Q., Luo D.-N. (2017). Correlations of Foxo3 and Foxo4 expressions with clinicopathological features and prognosis of bladder cancer. Pathol. Res. Pract..

[46] Tenbaum S.P., Ordóñez-Morán P., Puig I., Chicote I., Arqués O., Landolfi S., Fernández Y., Herance J.R., Gispert J.D., Mendizabal L. (2012). β-Catenin confers resistance to PI3K and AKT inhibitors and subverts FOXO3a to promote metastasis in colon cancer. Nat. Med..

[47] Yang X., Zhao J., Huang C., Wang Q., Pan K., Wang D., Pan Q., Jiang S., Lv L., Gao X. (2013). Decreased expression of the FOXO3a gene is associated with poor prognosis in primary gastric adenocarcinoma patients. PLoS ONE.

