# Correction: Fondevila et al. Association of FOXO3 Expression with Tumor Pathogenesis, Prognosis and Clinicopathological Features in Hepatocellular Carcinoma: A Systematic Review with Meta-Analysis. *Cancers* 2021, *13*, 5349

**DOI:** 10.3390/cancers17030350

**Published:** 2025-01-22

**Authors:** Flavia Fondevila, Paula Fernández-Palanca, Carolina Méndez-Blanco, Tania Payo-Serafín, Elisa Lozano, Jose J. G. Marin, Javier González-Gallego, José L. Mauriz

**Affiliations:** 1 Institute of Biomedicine (IBIOMED), University of León, Campus de Vegazana s/n, 24071 León, Spain; ffonp@unileon.es (F.F.); pferp@unileon.es (P.F.-P.); cmenb@unileon.es (C.M.-B.); tpayos00@estudiantes.unileon.es (T.P.-S.); jgonga@unileon.es (J.G.-G.); 2 Centro de Investigación Biomédica en Red de Enfermedades Hepáticas y Digestivas (CIBERehd), Instituto de Salud Carlos III, Av. de Monforte de Lemos 5, 28029 Madrid, Spain; elisa_biologia@usal.es (E.L.); jjgmarin@usal.es (J.J.G.M.); 3 Experimental Hepatology and Drug Targeting (HEVEPHARM), Salamanca Biomedical Research Institute (IBSAL), University of Salamanca, 37007 Salamanca, Spain

**References Updated**

In the original publication [1], Two references from other authors included in this article have been retracted since the publication of our manuscript. These references were not included in the meta-analysis study and originally appeared only in the Introduction and Discussion Sections. The scientific findings and conclusions are not affected. The authors apologize for any inconvenience. The retracted references have been updated in the original publication. This correction was approved by the Academic Editor. 

Ref. [17]: Yang, S.; Pang, L.; Dai, W.; Wu, S.; Ren, T.; Duan, Y.; Zheng, Y.; Bi, S.; Zhang, X.; Kong, J. Role of forkhead box O proteins in hepatocellular carcinoma biology and progression (Review). *Front. Oncol.* **2021**, *11*, 667730. https://doi.org/10.3389/fonc.2021.667730 **instead of** Liu, Z.; Li, Z.; Xu, B.; Yao, H.; Qi, S.; Tai, J. Long noncoding RNA PRR34-AS1 aggravates the progression of hepatocellular carcinoma by adsorbing microRNA-498 and thereby upregulating FOXO3. *Cancer Manag. Res.* **2020**, *12*, 10749–10762. https://doi.org/10.2147/CMAR.S263619.

Ref. [36]: Yu, S.; Yu, Y.; Zhang, W.; Yuan, W.; Zhao, N.; Li, Q.; Cui, Y.; Wang, Y.; Li, W.; Sun, Y.; et al. FOXO3a promotes gastric cancer cell migration and invasion through the induction of cathepsin L. *Oncotarget* **2016**, *7*, 34773–34784. https://doi.org/10.18632/oncotarget.8977 **instead of** Zhou, Y.; Chen, Y.; Ding, W.; Hua, Z.; Wang, L.; Zhu, Y.; Qian, H.; Dai, T. LncRNA UCA1 impacts cell proliferation, invasion, and migration of pancreatic cancer through regulating miR-96/FOXO3. *IUBMB Life* **2018**, *70*, 276–290. https://doi.org/10.1002/iub.1699.
